# RECRUITING, FACILITATING, AND SUPPORTING PERSONS LIVING WITH DEMENTIA AS TRAINERS IN RESEARCH

**DOI:** 10.1093/geroni/igac059.1280

**Published:** 2022-12-20

**Authors:** Jen Hirsch, Fei Sun, Dan Cohen, Ha-Neul Kim, Steven Barbieri, Brian LeBlanc

**Affiliations:** Michigan State University, East Lansing, Michigan, United States; Michigan State University, East Lansing, Michigan, United States; Right to Music, Mineola, New York, United States; Michigan State University, East Lansing, Michigan, United States; National Council of Dementia Minds, Alma, Michigan, United States; National Council of Dementia Minds, Alma, Michigan, United States

## Abstract

Research demonstrates that dementia education programs are effective in improving knowledge of dementia, but it takes more effort to change attitudes or skill sets. Empathy toward people living with dementia (PLWD) is low among college students due to lack of experience interacting with PLWD. Exposure to PLWD is recommended in training aimed to improve dementia literacy. We engaged PLWD from an active advocacy group, The National Council of Dementia Minds, to be co-trainers in an intervention with college students in health and social care professions to provide quality care for PLWD. Presenters will discuss the process for recruiting, engaging, and training PLWD successfully, including explanation of informed consent, preparation for training sessions, and structural support around communication and information processing needs. Emphasis will be on lessons learned directly from our co-trainers for successful support mechanisms, particularly in an online environment as all interaction occurred primarily over zoom and email.

